# Effects of Dynamic Light Regimes on Yield and Quality Properties of *Pleurotus pulmonarius* Cultivar ‘Jinxiu’

**DOI:** 10.3390/jof12060426

**Published:** 2026-06-11

**Authors:** Bin Yu, Jiling Song, Jiandong Lai, Shuting Xu, Weidong Yuan, Qing Chen

**Affiliations:** 1 Hangzhou Agricultural Technical Extension Center, Hangzhou 310016, China; hztinayu@126.com (B.Y.); xst951008@163.com (S.X.); 2 Hangzhou Academy of Agricultural Sciences, Hangzhou 310024, China; songjiling860605@163.com; 3 Hangzhou Guanxin Agricultural Science and Technology Co., Ltd., Hangzhou 311217, China; 13606610846@163.com; 4 Zhejiang Agricultural Technical Extension Center, Hangzhou 310020, China

**Keywords:** *Pleurotus pulmonarius*, light regimes, pre-illumination, LED lighting, fruiting body development, nutrient accumulation, yield, quality

## Abstract

Light is a critical environmental cue regulating development and quality in edible fungi, yet the effects of dynamic light regimes (for example, transitions from white to blue light) remain poorly understood. We systematically investigated how white-light pretreatment duration (0, 4, 8, or 12 h) and two blue-light regimes—B6 (6 h blue followed by white until harvest) and Bc (continuous blue until harvest)—affect fruiting-body development, yield, color, textural properties, and nutritional quality of *Pleurotus pulmonarius*. The experiment was conducted at a single commercial production facility in Zhejiang Province, China, using the commercial strain *P. pulmonarius* (cultivar ‘Jinxiu’). Two-way ANOVA revealed significant interactions between white-light pretreatment and blue-light regime for cap a* value (red-green), cap width, cap hardness and chewiness, stipe hardness, number of fruiting bodies, and several nutrient components. All dynamic light regimes reduced cap L* value (lightness) and b* value (yellow-blue); continuous blue (Bc) produced a darker cap. Yield responses to blue-light duration depended on pretreatment: without white pretreatment, Bc outperformed B6, whereas with 4–12 h white pretreatment B6 produced higher yields. Relative to the control (CK), all dynamic regimes significantly increased total free amino acids and essential amino acids. Except for W4B6 and W12B6, all other treatments significantly increased crude protein; total soluble sugar, crude fat, and crude fiber decreased in most treatments compared to CK. These results indicate that an optimized transition from white to blue light can synergistically improve the color, nutritional quality and yield of *P. pulmonarius*. The W8Bc regime (8 h white pretreatment followed by continuous blue until harvest) produced the highest cap chewiness (21.65 N·mm) and free amino acid content (3110.44 μg·g^−1^), the darkest cap color, and the top comprehensive score in the entropy-weighted TOPSIS evaluation, despite ranking second in yield and high-quality rate. Under the conditions tested (single cultivar ‘Jinxiu’ at one production base), we recommend the W8Bc light regime as suitable for industrial cultivation of *Pleurotus pulmonarius*. However, it should be noted that these findings cannot be generalized to the entire species without further validation across multiple strains and multiple locations.

## 1. Introduction

**Figure 1:**
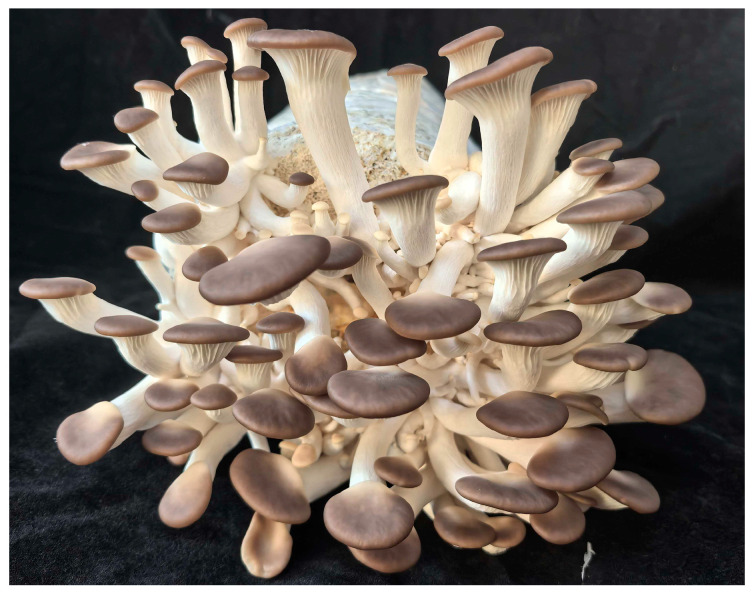
Growth of *P. pulmonarius*.

*Pleurotus pulmonarius* is a macrofungus valued for both its culinary and medicinal properties. Taxonomically, it belongs to the family Pleurotaceae (order *Agaricales*, *phylum Basidiomycota*) [1]. According to the China Edible Fungi Association, China produced 636,700 tons of *P. pulmonarius* in 2022 [2]; production subsequently increased markedly, rising 14.63% by 2024. Because of its crisp texture, high nutritional value [3,4], short cultivation cycle, and suitability for large-scale farming [1], *P. pulmonarius* has become a major industrial mushroom in China. Recent advances in industrialized cultivation have shifted production from seasonal farming to fully controlled, environmentally optimized systems that permit year-round cultivation (Figure 1) [5,6].

Despite these technological advances, precise control of environmental factors to optimize both yield and the quality of *P. pulmonarius* fruiting bodies remains a key scientific challenge for further improving production efficiency [7,8,9]. Light is an important environmental cue that regulates early differentiation, fruiting-body formation, and the biosynthesis of nutritional compounds in edible mushrooms [10,11,12]. Consequently, the effects of different light spectra on growth, development, and morphology in cultivated fungi have become an active area of research in recent years [13,14].

Previous studies indicate that blue light promotes cap differentiation and increases protein and amino acid contents in the fruiting bodies of several species, including *Flammulina filiformis* [10] and *Pleurotus eryngii* [12]. Mechanistic work in model fungi such as *Cordyceps militaris* is clarifying the roles of blue-light receptors (for example, the White Collar complex) and downstream regulatory networks in development and secondary metabolism [15,16]. However, most studies have focused on continuous exposure to monochromatic light. Far fewer investigations have examined the effects of dynamic light regimes—for example, combinations of spectra and fluctuating photoperiod durations—on agronomic performance and nutritional quality. The impacts of such complex light conditions are still poorly understood and deserve further study.

In plants, dynamic light signals—such as transitions between red and far-red light—are well established as environmental cues that activate complex signaling networks regulating morphogenesis and developmental transitions [17,18]. Accordingly, we designed dynamic light regimes that varied the duration of white-light pre-exposure (0, 4, 8, and 12 h) followed by two blue-light regimes: 6 h blue followed by white until harvest, or continuous blue until harvest. We systematically evaluated the effects of these treatments on yield, fruiting-body morphology, textural properties, and nutritional quality of *P. pulmonarius*. Two-way ANOVA was used to analyze the interaction between white-light pretreatment and blue-light regime. For multi-trait assessment and selection of the optimal light regime, we applied the entropy-weighted TOPSIS method, which has been widely employed for comprehensive quality evaluation of agricultural products [19,20,21].

The objectives of this study were to: (1) characterize variation patterns in yield and quality-related traits of *P. pulmonarius* in response to different light regimes; (2) identify the optimal combination of white-light pretreatment and blue-light regime using an entropy-weighted TOPSIS (Technique for Order of Preference by Similarity to Ideal Solution) multi-criteria evaluation; and (3) provide a theoretical basis and practical guidance for optimizing light environments in the industrial cultivation of edible fungi.

## 2. Materials and Methods

### 2.1. Materials and Reagents

The test strain was *Pleurotus pulmonarius* ‘Jinxiu’, a major cultivar in Zhejiang Province. The growth substrate consisted of 30% cottonseed hulls, 15% maize cobs, 28% wood shavings, 15% bran, 5% maize flour, 5% soybean meal, 1% lime, and 1% calcium bisulfate (percentages *w*/*w*). After thorough mixing, the substrate was packed into polyethylene bags (18.5 cm × 36 cm × 0.005 cm) at 1.25 kg wet weight per bag (moisture content 62%).

Most reagents—sulfuric acid (98%, GR), anthrone (AR), hydrochloric acid (37%, GR), potassium hydroxide (≥85%, AR), ethanol (≥99.7%, AR), boric acid (≥99.5%, AR), ammonium sulfate (≥99%, AR), copper sulfate (≥99%, AR), potassium sulfate (≥99%, AR), methyl red (indicator grade), and bromocresol green (indicator grade)—were supplied by Sinopharm Chemical Reagents Co., Ltd. (Shanghai, China). Glucose (≥99.5%, HPLC grade) was obtained from Sigma (St. Louis, MO, USA). Anhydrous diethyl ether (≥99.5%, AR), petroleum ether (boiling range 60–90 °C, AR), methanol (≥99.9%, HPLC grade), and formic acid (≥98%, AR) were obtained from ANPEL Laboratory Technologies (Shanghai) Inc. (Shanghai, China). All reagents were used as received without further purification; their purity was verified by the suppliers’ certificates of analysis.

### 2.2. Instruments and Equipment

The following instruments were used: Texture analyser (FTC tms-plus, Zhejiang TopCloud Agricultural Technology Co., Ltd., Hangzhou, China); Colorimeter (YS3060, Shenzhen ThreeNH Technology Co., Ltd., Shenzhen, China); Electronic balance (ESJ30-5A, Shenyang Shenyu Longteng Balance Co., Ltd., Shenyang, China); Muffle furnace (SX-4-10, Shanghai Techeng Machinery Equipment Co., Ltd., Shanghai, China); Rapid moisture analyser (HR83-P, Mettler Toledo Inc., Greifensee, Switzerland); Fully automatic Kjeldahl nitrogen analyser (K-370, BUCHI Labortechnik AG, Flawil, Switzerland); Digestion unit (S402, Shandong Haineng Instrument Co., Ltd., Jinan, China); Low-temperature high-speed centrifuge (5430R, Eppendorf AG, Hamburg, Germany); Tissue homogeniser (Tissuelyser-48, Shanghai Jingxin Industrial Development Co., Ltd., Shanghai, China); Multifunctional microplate reader (Multiskan GO), ultra-high-performance liquid chromatograph (Vanquish) and mass spectrometer (Q Exactive), all purchased from Thermo Fisher Scientific Inc., Waltham, MA, USA.

### 2.3. Experimental Design and Methods

The experiment was conducted at the Tongxiang Xi’an Mushroom Industry Cooperative (Tongxiang, China). A liquid inoculum was used to ensure uniform inoculation. After 60 days of incubation at room temperature, the cultures were transferred to an automated, controlled-environment fruiting chamber at Hangzhou Guanxin Agricultural Technology Co., Ltd. (Hangzhou, China) for fruiting. Germination and primordium formation were managed according to standard procedures. When fruiting bodies reached the shaping stage and received a single heavy watering, we applied eight dynamic light regimes and one continuous white-light control (CK). Each treatment consisted of a white-light pretreatment (0, 4, 8, or 12 h) followed by one of two blue-light regimes: (1) 6 h of blue light then continuous white light until harvest (B6), or (2) continuous blue light until harvest (Bc). The specific combinations are summarized in Table 1.

The substrate bags of the nine treatments were then arranged on shelves within the chamber, with three replicates per treatment and 100 bags per replicate (total of 2700 bags). The shelves were placed against the wall, and each bag was positioned with its fruiting surface facing outward (parallel to the wall). Blue and white LED light strips were installed directly facing the fruiting surface at a distance of 100 cm. The spectral characteristics were measured using a SPIC-500AW Spectral Colorimeter (EVERFINE Corporation, Hangzhou, China). For the white LED: correlated color temperature 10,714 K, peak wavelength 446.3 nm, dominant wavelength 477.0 nm, color purity 23.3%, luminous flux 11.03 lm, luminous efficacy 199.84 lm/W. For the blue LED: correlated color temperature 100,000 K, peak wavelength 464.7 nm, dominant wavelength 468 nm, color purity 93.3%, luminous flux 7.99 lm, luminous efficacy 47.09 lm/W. Both LEDs were set to an illuminance of approximately 390 lx. Racks for different treatments were fully separated with blackout fabric to prevent light contamination between treatments (Figure S1). The entire experiment was conducted in three independent temporal blocks (i.e., three consecutive cultivation cycles). Environmental parameters included temperature (23 ± 1 °C), relative humidity (85–90%), CO_22_ concentration (800–1200 ppm), and airflow rate (0.5–1.0 m/s). During the experiment, fruiting characteristics and yield were recorded for each replicate. Because preliminary analyses indicated no significant effect of block on the measured parameters, the three temporal blocks were treated as replicates for statistical analysis.

### 2.4. Indicators and Methods for Determination

#### 2.4.1. Measurement of Agronomic Traits

Fruiting bodies are ready for harvest when mature, characterized by a deep-gray cap (3–5 cm in diameter) and a pure-white stem (3–6 cm long, 1–2 cm in diameter). From each treatment, 30 fruiting bodies were randomly selected and measured with vernier calipers for cap length, cap width, cap thickness, stipe length, and stipe diameter. After harvesting each plot, total yield was recorded and average yield per bag calculated. The high-quality rate (the weight ratio of Grade A fruiting bodies to total yield) were determined following Rao et al. [22]. Additionally, 15 substrate bags per treatment were randomly selected and the number of fruiting bodies per bag was recorded. The harvest period was defined as the interval from the commencement of cold stimulation to completion of harvesting for a single flush. From the same 30 fruiting bodies, nine were randomly selected for color measurement. Color (L* for lightness, a* for red–green, and b* for yellow–blue) was measured at the center of each cap using a colorimeter, with nine replicates per treatment (*n* = 9).

#### 2.4.2. Characterization of Texture

The textural properties of the fruiting bodies of *P. pulmonarius* were examined using a texture analyzer. The specific parameters were determined with reference to the previous literature [23].

#### 2.4.3. Determination of Nutrient Content

The nutritional composition of *P. pulmonarius* fruiting bodies was determined following Chinese national standards: GB/T 15672-2009 [24] (Determination of total saccharides in edible mushrooms), GB/T 6438-2022 [25] (Determination of crude ash in feeds), GB/T 6433-2006 [26] (Determination of crude fat in feeds), GB/T 5009.10-2003 [27] (Determination of crude fiber in vegetable foods), GB/T 6432-2018 [28] (Determination of crude protein in feeds—Kjeldahl method), and GB/T 30987-2020 [29] (Determination of free amino acids in plants).

#### 2.4.4. Comprehensive Evaluation by the Entropy-Weighted TOPSIS Method

We selected agronomic traits, yield, and nutritional-quality indicators of *P. pulmonarius* fruiting bodies exposed to nine distinct light regimes. Indicator weights were computed using the entropy-weighted method, and the relative closeness of each treatment to the ideal solution was calculated using the Technique for Order Preference by Similarity to Ideal Solution (TOPSIS) to produce a comprehensive ranking [30]. First, an evaluation matrix X*_ij_* (*i* = 1, 2, …, *n*; *j* = 1, 2, …, *m*; n = 9, m = 25) was constructed for the nine light regime and the selected indicators.

For “larger is better” indicators, we applied the following normalization: ${X^{\prime }}_{\mathrm{ij}}=\frac{X_{\mathrm{ij}}-\min(X_{j})}{\max(X_{j})-\min(X_{j})}$; for “smaller is better” indicators—such as L*, a*, and b* values and crude fat content—we applied the following normalization: ${X^{\prime }}_{\mathrm{ij}}=\frac{\max(X_{j})-X_{\mathrm{ij}}}{\max(X_{j})-\min(X_{j})}$. To prevent zeros in subsequent entropy calculations, we shift all normalized data to nonnegative values using Formula ${X^{\prime \prime }}_{\mathrm{ij}}={X^{\prime }}_{\mathrm{ij}}+0.001$. We then compute the information entropy (e_j_) and the corresponding weight (W_j_) for each indicator as follows:

(1) $$P_{\mathrm{ij}}=\frac{{X^{\prime \prime }}_{\mathrm{ij}}}{\sum_{i=1}^{n}{X^{\prime \prime }}_{\mathrm{ij}}};$$

(2) $$e_{j}=-k\sum_{i=1}^{n}P_{\mathrm{ij}}\ln(P_{\mathrm{ij}});$$

(3) $$k=\frac{1}{\ln(n)};$$

(4) $$W_{j}=\frac{1-e_{j}}{\sum_{j=1}^{m}\left(1-e_{j}\right)};$$

Multiply each normalized indicator value by its corresponding weight to construct the weighted decision matrix Vij(*i* = 1, 2,……*n*; *j* = 1, 2,……*m*). Determine the positive ideal solution $V^{+}=\left(\max V_{i1},\;\max V_{i2},\;\ldots ,\;{\mathrm{maxV}}_{\mathrm{im}}\right)$ and the negative ideal solution $V^{-}=\left(\min V_{i1},\;\min V_{i2}\;\ldots ,\;{\mathrm{minV}}_{\mathrm{im}}\right)$. Compute the distance D*_i_*^+^ from each treatment to the positive ideal solution and the distance D*_i_*^−^ from each treatment to the negative ideal solution. Finally, calculate the relative closeness C*_i_* for each treatment, where C*_i_* ranges from 0 to 1 and higher values indicate superior overall performance.

(5) $$D_{i}^{+}=\sqrt{\sum_{j=1}^{m}{\left(V_{\mathrm{ij}}-V_{j}^{+}\right)}^{2}}$$

(6) $$D_{i}^{-}=\sqrt{\sum_{j=1}^{m}{\left(V_{\mathrm{ij}}-V_{j}^{-}\right)}^{2}}$$

(7) $$C_{i}=\frac{D_{i}^{-}}{D_{i}^{+}+D_{i}^{-}}$$

#### 2.4.5. Statistical Analysis

Data were organized in Microsoft Excel 2010, and entropy-weighted TOPSIS analysis was performed in Excel. Statistical analyses were conducted in SPSS version 25.0. First, a two-way ANOVA was applied to the eight light regimes; when a significant interaction was detected, simple effects analyses were performed. Subsequently, all treatment groups and the control (CK) were analyzed by one-way ANOVA, followed by Dunnett’s test to compare each treatment with the control (*p* < 0.05). Figures were produced with GraphPad Prism version 10.1.2.

## 3. Results and Analysis

### 3.1. Effects of Light Regimes on the Color and Morphology of P. pulmonarius Fruiting Bodies

**Figure 2:**
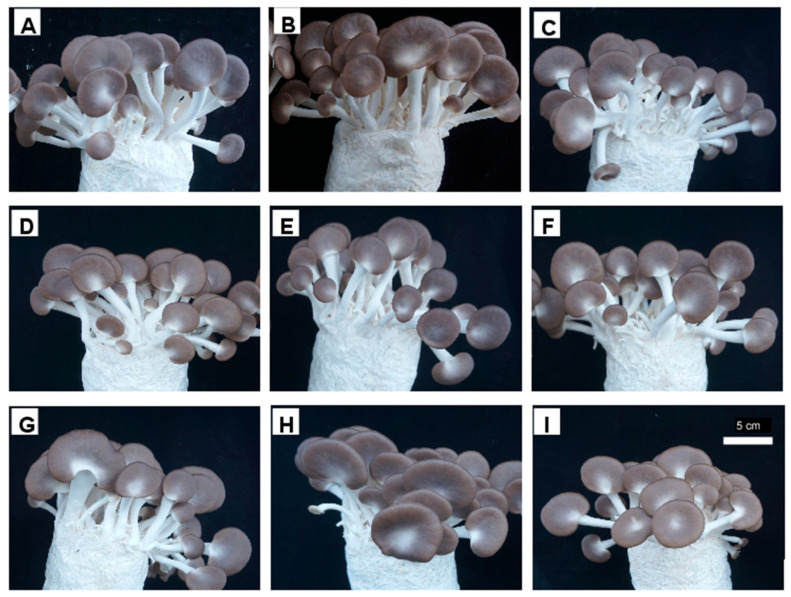
Growth of *P. pulmonarius* under different light-regime treatments. (**A**): W0B6; (**B**): W0Bc; (**C**): W4B6; (**D**): W4Bc; (**E**): W8B6; (**F**): W8Bc; (**G**): W12B6; (**H**): W12Bc; (**I**): CK.

**Figure 3:**
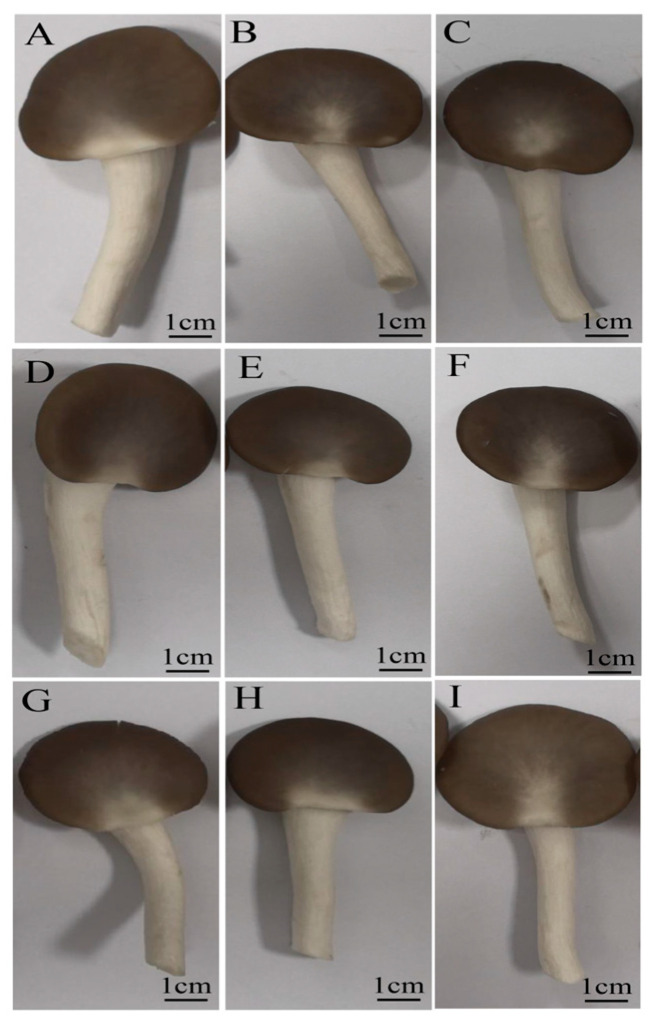
Appearance of individual *P. pulmonarius* under different light-regime treatments. (**A**): W0B6; (**B**): W0Bc; (**C**): W4B6; (**D**): W4Bc; (**E**): W8B6; (**F**): W8Bc; (**G**): W12B6; (**H**): W12Bc; (**I**): CK.

**Figure 4:**
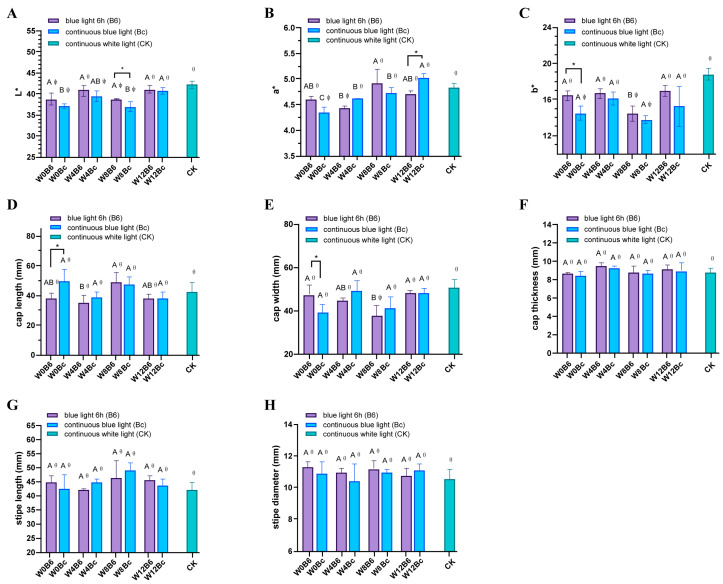
Effects of light regimes on the color and morphology of *P. pulmonarius* fruiting bodies. (**A**) L*; (**B**) a*; (**C**) b*; (**D**) cap length; (**E**) cap width; (**F**) cap thickness; (**G**) stipe length; (**H**) stipe diameter. Data are presented as mean ± SEM (*n* = 3). Purple represents treatments with 6 h blue light followed by white until harvest (B6). Blue represents treatments with continuous blue light until harvest (Bc). Green indicates the continuous white-light control (CK). Statistical significance is indicated as follows: Different uppercase letters within the same blue-light duration group denote significant differences among white-light pretreatment durations (two-way ANOVA followed by Tukey’s HSD test, *p* < 0.05). * indicates significant differences between the two blue-light durations (6 h vs. continuous) within the same white-light pretreatment duration group (simple effect analysis with Bonferroni adjustment, *p* < 0.05). Different Greek letters (θ and ψ) indicate significant differences between the treatment groups and CK (one-way ANOVA followed by Dunnett’s test, *p* < 0.05).

As shown in Figure 2, Figure 3 and Figure 4A–C, the cap color was darker in all light-regime treatments than in the continuous white-light control (CK). Two-way ANOVA (Table 2) indicated that white-light pretreatment duration had highly significant effects on L*, a*, and b* (*p* < 0.01), whereas blue-light irradiation duration significantly affected L* and b* but not a*. The interaction between the two factors was significant only for a* (*F* = 4.33, *p* < 0.05). The main effect of blue light most strongly influenced L* and b* (*F* = 8.21 and 8.64, respectively; *p* < 0.05), while a* was most affected by the main effect of white light (*F* = 7.27; *p* < 0.01) and by the interaction (*F* = 4.33; *p* < 0.05). Relative to CK, L* decreased significantly in most treatments except W4B6, W12B6, and W12Bc (Figure 4A). For a given white-light pretreatment, L* values in Bc treatments were consistently lower than in the corresponding B6 treatments; for example, W8Bc (36.90) was significantly lower than W8B6 (38.77) by 4.82%, indicating that prolonged blue-light exposure after white-light pretreatment further deepens cap color. The a* value was significantly lower than CK only in W0Bc and W4B6, with reductions of 0.49 and 0.41, respectively (Figure 4B). The b* value was significantly lower than CK only in W0Bc, W8B6, and W8Bc, decreasing by 4.27, 4.37, and 5.01, respectively (Figure 4C). These results indicate that white-light pretreatment has a relatively limited effect on the red–green (a*) and yellow–blue (b*) color components of *P. pulmonarius* caps.

In terms of morphological characteristics, a two-way ANOVA (Table 2) indicated that the duration of white-light pretreatment was the primary factor affecting cap dimensions (length, width, and thickness; *p* < 0.05), whereas the duration of blue-light irradiation had no significant main effect on any morphological parameter (Figure 4D–H). The interaction between these two factors was significant only for cap width (*F* = 3.45, *p* < 0.05). White-light pretreatment exerted the strongest main effects on cap length (*F* = 6.18) and cap width (*F* = 5.97). Compared with the control (CK), only the W8B6 treatment produced a significant reduction in cap width (3.74 cm), a decrease of 26.1% (Figure 4E). Although cap length in W0Bc, W8B6, and W8Bc exceeded that of the control and cap thickness was greatest in W4B6 (9.53 mm), these differences were not statistically significant. No treatment differed significantly from the control in stipe length or diameter. Overall, the light regime produced only modest changes in fruiting-body morphology of *P. pulmonarius*, with the primary effects observed on color, notably reductions in L* and b* values.

### 3.2. Effects of Light Regimes on Yield and Textural Properties

**Figure 5:**
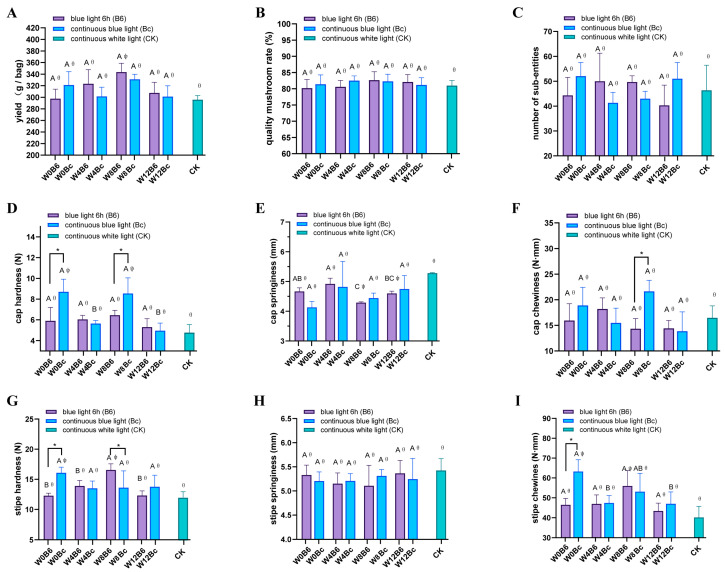
Effects of light regimes on yield and textural properties of *P. pulmonarius.* (**A**) Yield per bag; (**B**) percentage of high-quality fruiting bodies; (**C**) number of fruiting bodies; (**D**) cap hardness; (**E**) cap springiness; (**F**) cap chewiness; (**G**) stipe hardness; (**H**) stipe springiness; (**I**) stipe chewiness. Light regime treatments, data presentation, color coding, and statistical symbols are the same as described in Figure 4.

Different dynamic light-regime treatments significantly affected yield per bag (Figure 5A). A two-way ANOVA (Table 3) showed that the duration of white-light pretreatment was the primary factor influencing yield per bag (*F* = 3.90, *p* < 0.05), while the duration of blue-light exposure and its interaction with white light did not significantly affect yield. Although the interaction term was significant for the number of fruiting bodies (*F* = 3.30, *p* < 0.05), subsequent pairwise comparisons revealed no significant differences among treatments or between treatments and the control (CK) (Figure 5C). Among all treatments, only W8B6 produced a significant 16.1% increase in yield per bag (343.43 g/bag) relative to CK (Figure 5A). There were no significant differences in the percentage of high-quality fruiting-body rate (Figure 5B) across treatments, indicating that the dynamic light regimes tested had little effect on the commercial appearance of fruiting bodies.

Dynamic light regimes significantly improved textural characteristics, primarily increasing hardness and chewiness (Figure 5D–I). Two-way ANOVA (Table 3) showed that the duration of white-light pretreatment significantly affected cap hardness and stipe chewiness, whereas the duration of blue-light irradiation significantly affected only cap hardness. The interaction between the two factors significantly influenced cap hardness (*F* = 4.63, *p* < 0.05), cap chewiness (*F* = 3.74, *p* < 0.05), and stipe hardness (*F* = 5.99, *p* < 0.01). Compared with CK, W0Bc increased cap hardness (+83.4%), stipe hardness (+34.0%), and stipe chewiness (+57.3%); W8B6 increased stipe hardness (+37.9%) and stipe chewiness (+39.3%) but decreased cap springiness (−18.8%); W8Bc increased cap hardness (+80.0%); and W12B6 reduced cap springiness (−12.9%). These results indicate that continuous blue-light irradiation, or blue-light irradiation following a white-light pretreatment of specific duration (e.g., 8 h), can effectively enhance the hardness and chewiness of *P. pulmonarius* fruiting bodies, thereby extending their postharvest shelf life.

Overall, an 8 h white-light pretreatment followed by blue-light exposure produced superior outcomes, increasing yield per bag, the proportion of high-quality fruiting-body rate, and key textural properties (e.g., hardness and chewiness). These results indicate that appropriate light regimes can synergistically enhance both yield and texture of *P. pulmonarius*.

### 3.3. Nutritional Composition of P. pulmonarius Under Different Light Regimes

Dynamic light regimes had multifaceted and significant effects on the nutritional composition of *P. pulmonarius* fruiting bodies. Two-way analysis of variance (ANOVA; Table 4) showed that the duration of white-light pretreatment, the duration of blue-light irradiation, and their interaction all exerted highly significant effects (*p* < 0.01) on total soluble sugars, ash content, crude fat, crude fiber, crude protein, total essential amino acids, and total free amino acids. Ash content (*F* = 1333.39), crude fat (*F* = 1019.86), crude fiber (*F* = 5324.80), and crude protein (*F* = 1133.85) were primarily affected by the duration of white-light pretreatment, whereas the total contents of essential amino acids and free amino acids were especially influenced by the interaction between white- and blue-light durations (*F* = 2378.11 and *F* = 1821.79, respectively). Moisture content was significantly affected only by the main effects of white- and blue-light durations.

**Figure 6:**
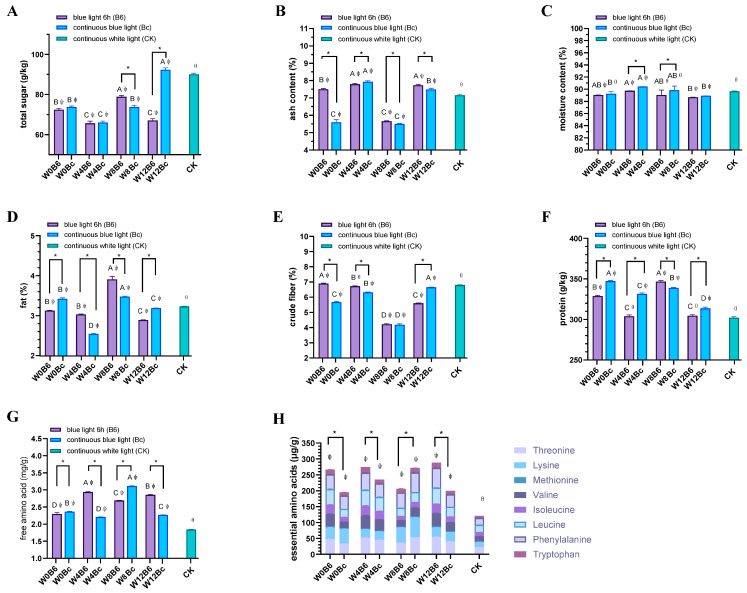
Effects of light regimes on nutritional quality of *P. pulmonarius.* (**A**) Total soluble sugar content; (**B**) ash content; (**C**) moisture content; (**D**) crude fat content; (**E**) crude fiber content; (**F**) crude protein content; (**G**) free amino acid content; (**H**) total essential amino acid composition. Light regime treatments, data presentation, color coding, and statistical symbols are the same as described in Figure 4.

Compared with the continuous white-light control (CK; Figure 6), total soluble sugar content decreased significantly in all treatments by 11.23–24.41 g·kg^−1^, except for W12Bc, which increased by 2.27 g·kg^−1^ (Figure 6A). Ash content responses were more complex: W0Bc, W8B6, and W8Bc showed significant decreases of 1.50–1.66 g·100 g^−1^, whereas the remaining treatments exhibited significant increases of 0.34–0.78 g·100 g^−1^ (Figure 4B). Moisture content varied by treatment: W0B6, W12B6, and W12Bc decreased significantly by 0.65–1.02 percentage points, while W8B6 and W8Bc increased by 0.06 and 0.75 percentage points, respectively (Figure 6C). Crude fat and crude fiber declined significantly in most treatments; the largest reduction in crude fat occurred in W4Bc (−0.69%), and the greatest reductions in crude fiber occurred in W8B6 and W8Bc (−2.58% and −2.61%, respectively) (Figure 6D,E). Crude protein content exceeded that of CK in all treatments, with significant increases in most treatments except W4B6 and W12B6 (Figure 6F). The most consistent and pronounced effect was on amino acid accumulation: all light regimes significantly increased total essential amino acids (by 74.12~166.94 μg·g^−1^) and total free amino acids (by 367.12~1269.14 μg·g^−1^) in the fruiting bodies (Figure 4G,H).

Different dynamic light regimes produced distinct directions and magnitudes of nutrient regulation. The W4Bc treatment produced the largest increase in crude protein and amino acid content while causing the greatest reduction in crude fat. The W0B6 treatment also showed high protein and amino acid levels and low fat, and it additionally caused a significant increase in crude fiber. The W8Bc treatment yielded the highest accumulation of free amino acids (3110.44 μg·g^−1^) of all treatments. These results indicate that the nutritional profile of *P. pulmonarius* can be selectively optimized by designing appropriate light regimes.

### 3.4. Comprehensive Ranking of Light Regimes Based on Entropy-Weighted TOPSIS Analysis

Using the entropy-weighted TOPSIS method, we analyzed 25 indicators—including agronomic traits, yield, and nutritional quality—of *P. pulmonarius* across nine dynamic light regimes and computed the information entropy (e) and corresponding weights (W) for each indicator. As shown in Table 5, indicators with greater variability received higher weights. The highest-weighted indicators were total soluble sugars (6.6%), yield (5.8%), stipe length (5.6%), cap hardness (5.5%), cap chewiness (5.5%), crude protein (5.3%), and stipe hardness (5.3%), while essential amino acids had the lowest weight (2.2%).

We then calculated the distance to the positive ideal solution (D+), the distance to the negative ideal solution (D−), and the relative closeness (C) for each treatment. As shown in Table 6, the W8Bc treatment (8 h of white light + blue light until harvest) exhibited the best overall performance (*C* = 0.466, ranked 1st), followed by W8B6 (8 h of white light + 6 h of blue light followed by white light until harvest, *C* = 0.446) and W0Bc (0 h of white light + blue light until harvest, *C* = 0.396). The CK (continuous white-light control) had the lowest overall score (*C* = 0.227, ranked 9th). Therefore, W8Bc was identified as the optimal regime treatment in this study and can be used in subsequent metabolomic and transcriptomic analyses to investigate the molecular mechanisms underlying its superior performance.

## 4. Discussion

### 4.1. Effects of Light Regimes on Cap Color

All eight dynamic light regimes significantly reduced cap lightness (L*) and the yellow–blue axis value (b*) of *P*. *pulmonarius* fruiting bodies; W0Bc, W8B6, and W8Bc produced the greatest darkening (Figure 4A,C). Blue-light-induced darkening of fruiting bodies has been reported for *Pleurotus citrinopileatus* [11], *Lyophyllum decastes* [14], *Morchella sextelata* [31], *Flammulina velutipes* [32], a pattern consistent with activation of melanin biosynthesis. In Lyophyllum decastes, blue light not only darkened cap color but also upregulated blue-light receptor genes (WC-1, WC-2, and Cry-DASH), implicating these photoreceptors in pigment regulation [14]. Similarly, in *Auricularia heimuer*, high light intensity increases melanin synthesis by upregulating melanin-related genes—such as tyrosinases (e.g., TYR1) and laccases (e.g., LAC1)—via activation of blue-light receptors, thereby intensifying fruiting-body color [33]. Transcriptomic analysis in Lentinula edodes also showed that blue light induces expression of genes potentially linked to pigment development, including an FAD–NAD-binding domain-containing protein [34]. The decreases in L* and b* observed here may therefore reflect blue-light-mediated upregulation of melanin- or tyrosinase-related genes, a hypothesis that should be tested in future experiments.

For a given white-light pretreatment duration, continuous blue light (Bc) produced a darker cap color than the regime that returned to white light after 6 h of blue light (B6). This finding is consistent with the significant main effect of blue-light duration on L* and b* values in the two-way ANOVA, indicating that prolonged blue-light exposure promotes color darkening independently of pretreatment duration. Although the interaction between white-light pretreatment duration and blue-light regime was not significant for L* and b*, the magnitude of color deepening appeared to vary with pretreatment duration. These results imply that blue-light-induced darkening is consistent in direction, while its magnitude can be modulated by selecting appropriate light regimes to optimize pigment accumulation.

### 4.2. Effects of Light Regimes on Yield and Its Condition-Dependent Response

Yield responses varied with blue-light duration. Without white-light pretreatment (0 h), yield under continuous blue light (W0Bc) exceeded that under a regime of 6 h of blue light followed by white-light (W0B6). By contrast, when white-light pretreatment lasted 4, 8, or 12 h, the 6 h blue-light treatment (B6) generally produced higher yields than continuous blue light (Bc) (Figure 5A). Although the interaction between pretreatment duration and blue-light regime did not reach statistical significance (Table 3; *F* = 1.76, *p* = 0.195), these contrasting patterns suggest that the optimal blue-light strategy depends on whether white-light pretreatment is applied. A possible explanation, is that white-light pretreatment may alter the mycelium’s subsequent response to blue light [35,36,37]. Similar phenomena have been reported in other fungi. For example, in Metarhizium, brief light exposure induces tolerance to subsequent UV-B radiation [38]; in Monilinia fructicola: blue-light receptor genes are upregulated within minutes of illumination [39]; and in Metarhizium robertsii, white and blue light upregulate stress-responsive genes [40]; and epigenetic mechanisms such as DNA methylation and histone modifications have been shown to mediate fungal adaptation to environmental change [41]. Based on these reports, we hypothesize that white-light pretreatment in *P. pulmonarius* may prime the mycelium—potentially via transcriptional or epigenetic modifications—thereby modulating the subsequent growth-promoting effects of blue light. However, this hypothesis remains to be tested by molecular analyses (e.g., transcriptomics, qPCR, or epigenomic profiling) in future studies. From a production standpoint, following an 8 h white-light pretreatment, the blue-light regimes W8B6 and W8Bc increased yield by 16.1% and 11.9%, respectively, compared to the continuous white-light control (CK). These two regimes are therefore recommended for industrial cultivation of *P. pulmonarius*.

### 4.3. Effects of Light Regimes on Textural Properties

Improvements in textural properties arose from specific combinations of white-light duration and blue-light modality. Cap hardness increased primarily in response to continuous blue light (e.g., following either no white-light pretreatment or an 8 h white-light pretreatment). In contrast, changes in stipe hardness and chewiness were more complex. Under the B6 treatment, an 8 h white-light pretreatment significantly increased stipe hardness and chewiness; under the Bc treatment, the same 8 h white-light pretreatment decreased these traits. A two-way ANOVA detected a highly significant interaction for stipe hardness (*p* < 0.01) and a marginal interaction for stipe chewiness (*p* = 0.051). These opposing effects clearly indicate that cap and stipe respond differently to light signals.

Mushroom hardness is known to be related to cell-wall chitin and β-glucan content [42]. Previous studies have shown that blue light can upregulate expression of cell-wall-related enzymes, including β-glucan synthase and chitinase, via photoreceptors such as WC, thereby affecting cell-wall structure and fruiting-body morphogenesis [34]. Transcriptomic analyses in *Lentinula edodes* have also revealed blue-light-induced upregulation of genes involved in cell-wall structure and assembly [34]. Accordingly, it is plausible that the increased cap hardness observed in the W0Bc and W8Bc treatments is associated with altered cell-wall properties [43], although direct evidence (e.g., cell-wall composition analysis or gene expression profiling) is needed to confirm this association.

From a production standpoint, enhanced hardness and chewiness contribute to improved storage and transport stability, as well as an extended shelf life for fruiting bodies. This study is the first to systematically document the influence of light regime shifts on the textural characteristics of *P. pulmonarius*, highlighting the interplay between white-light pretreatment duration and blue-light regime in determining texture.

### 4.4. Effects of Light Regimes on Nutritional Quality

The results indicate that white-light pretreatment duration, the blue-light irradiation regime, and their interaction significantly affected all measured nutritional-quality parameters of *P. pulmonarius.* Across treatments, a consistent pattern was observed: all dynamic regimes increased crude protein and amino acids compared to CK, while crude fat, total soluble sugars, and crude fiber generally decreased (Figure 6). This finding aligns with a previous review indicating that blue light may enhance nutritional value by redirecting metabolic flux toward nitrogen assimilation [44].

The W8Bc treatment, which ranked first in the TOPSIS evaluation, produced the highest free amino acid content (68.9% above CK). A similar effect has been reported in *Pleurotus ostreatus*, where blue light activates amino acid biosynthesis [45]. Under 8 h white-light pretreatment, the W8B6 treatment exhibited significantly higher crude protein (346.6 g·kg^−1^), essential amino acids (206.5 μg·g^−1^), and free amino acids (2690.3 μg·g^−1^) than the CK (Figure 6F–H). However, compared with W8Bc, W8B6 showed a 12.5% increase in crude fat (Figure 6D). This difference suggests that the duration of blue-light exposure may influence carbon partitioning between protein/lipid biosynthetic pathways [45]. Specifically, reverting to white light after 6 h of blue light (B6) appeared to favor lipid accumulation, whereas continuous blue light (Bc) seemed to bias metabolism toward nitrogen assimilation. Comparison of W8Bc and W0Bc showed that W0Bc had higher crude protein but lower free amino acids than W8Bc, suggesting that the 8 h white-light pretreatment may influence how nitrogen is allocated between structural proteins and the free amino acid pool. These interpretations, however, are descriptive and should be treated as hypotheses to be tested by future metabolomin and transcriptomic analyses.

Other regimes also offer tailored nutritional profiles. For instance, W4Bc exhibited the lowest crude fat while retaining high protein and essential amino acids, suitable for low-fat products; W0B6 showed the highest crude fiber, with potential for functional foods targeting gut health [46]. Thus, by selecting appropriate light regime transitions, the nutritional composition of *P. pulmonarius* can be adjusted to meet specific market demands.

These light-driven nutritional modifications have direct implications for human health. As reviewed by Feng et al. [44], light regimes can enhance bioactive compounds that reduce chronic disease risk. W8Bc-grown mushrooms are richer in protein and amino acids, supporting muscle and metabolic health. W4Bc provides a low-fat option aligned with cardiovascular recommendations, while W0B6 offers high-fiber prebiotic effects for gut health [46]. Thus, light-regime adjustments can be used to tailor *P. pulmonarius* to market preferences and to specific health-oriented applications [44,46].

To our knowledge, this is the first systematic study of how light regime transitions affect the nutritional quality of this species. Future multi-omics studies are needed to elucidate the underlying regulatory networks [44].

### 4.5. Effects of Light Regimes on Comprehensive Performance of P. pulmonarius

The entropy-weighted TOPSIS evaluation indicated that treatment W8Bc achieved the best overall performance, followed by W8B6 and W0Bc. W8Bc’s overall advantage derives from superior values for yield (331.07 g·bag^−1^), cap chewiness (21.65 N·mm), cap hardness (8.55 N), protein content (338.99 g·kg^−1^), and free amino acids (3110.44 μg·g^−1^). Concurrent reductions in total sugars and crude fiber support the carbon–nitrogen metabolic trade-off described above. The second-ranked W8B6 shared the 8 h white-light pretreatment with W8Bc but differed in blue-light regime; both treatments produced significant increases in protein and amino acid contents, while W8B6 showed a pronounced increase in fat content, further indicating that blue-light duration regulates carbon metabolic flux. This study represents the first application of entropy-weighted TOPSIS to optimize and screen light regimes for *P. pulmonarius*, offering a more comprehensive and objective evaluation than single-indicator assessments. Accordingly, W8Bc is a promising dynamic light regime for industrial production of *P. pulmonarius*; its underlying molecular mechanisms merit further investigation using subsequent omics studies.

### 4.6. Limitations of the Study

It should be acknowledged that the present study was conducted using only a single commercial cultivar of *P. pulmonarius* (‘Jinxiu’), which is the predominant strain currently used in industrialized cultivation in Zhejiang Province, China. Responses to dynamic light regimes may be genotype-dependent, as different strains could exhibit variation in photosensitivity, metabolic flux regulation, and downstream gene expression patterns. Therefore, the findings reported here—while robust for the ‘Jinxiu’ cultivar under the tested conditions—cannot be safely generalized to the entire species *P. pulmonarius* without further validation across multiple genetically distinct strains. Future studies should aim to include a broader range of strains to assess the universality of the observed light-induced effects and to establish genotype-specific light management strategies for industrial production.

## 5. Conclusions

This study clarified how white-light pretreatment duration and blue-light regimes regulate the yield and quality of *P. pulmonarius* fruiting bodies. Dynamic light regimes significantly affected fruiting-body color, texture, and nutritional composition, revealing a clear trade-off between carbon- and nitrogen-based metabolism: protein and amino acid concentrations generally increased, whereas carbon-based constituents (e.g., sugars and fiber) declined. The W8Bc treatment (8 h white-light pretreatment followed by continuous blue-light irradiation until harvest) was the most effective overall, demonstrating the darkest cap color, along with improvements in hardness, proportion of high-quality fruiting bodies, yield, and protein and amino acid contents. Under the conditions tested (single cultivar ‘Jinxiu’, one production base), this treatment can serve as an optimized light regime for industrial-scale *P. pulmonarius* production and provides a theoretical foundation for the precise regulation of light conditions in edible-fungi cultivation. It should be noted that the above conclusions require further validation under conditions involving multiple strains and multiple locations.

From a consumer perspective, light management during fruiting can enhance the nutritional value of *P. pulmonarius* without genetic modification. *P. pulmonarius* grown under the W8Bc regime are richer in protein and free amino acids, supporting muscle maintenance and metabolic health. The W4Bc regime produces a low-fat product that may help manage cardiovascular risk, while W0B6 yields a high-fiber, prebiotic option that supports gut health. Although these results derive from a single cultivar, they suggest that dynamic light regimes could allow consumers to select mushrooms tailored to specific dietary goals. Translation of these findings by nutritionists and food scientists is needed.

## Figures and Tables

**Table 1:** Specific settings of the light regimes.

Formula		Treatments
B6	W0B6	No white pretreatment → 6 h blue light → white light until harvest
	W4B6	4 h white light → 6 h blue light → white light until harvest
	W8B6	8 h white light → 6 h blue light → white light until harvest
	W12B6	12 h white light → 6 h blue light → white light until harvest
Bc	W0Bc	No white pretreatment → continuous blue light until harvest
	W4Bc	4 h white light → continuous blue light until harvest
	W8Bc	8 h white light → continuous blue light until harvest
	W12Bc	12 h white light → continuous blue light until harvest
CK		Continuous white light until harvest

**Table 2:** Two-way ANOVA (F-values) of the effects of white-light pretreatment and blue-light irradiation on the color and morphology of *P. pulmonarius* fruiting bodies.

Source	L	a	b	CL	CW	CT	SL	SD
White	5.021 *	7.273 **	6.352 **	6.181 **	5.969 **	3.447 *	2.392	0.624
Blue	8.209 *	0.902	8.637 *	2.676	0.001	1.023	0.049	0.693
W × B	0.023	4.325 *	0.772	1.841	3.451 *	0.014	1.030	0.628

Note: L. cap L* value; a. cap a* value; b. cap b* value; CL. cap length; CW. cap width; CT. cap thickness; SL. stipe length; SD. stipe diameter; * indicating significant difference level (*p* < 0.05). ** indicating significant difference level (*p* < 0.01).

**Table 3:** Two-way ANOVA (F-values) of the effects of white-light pretreatment and blue-light irradiation on yield and textural properties of *P. pulmonarius* fruiting bodies.

Source	Yield	NSE	QR	CH	CS	CC	SH	SS	SC
White	3.903 *	0.191	0.522	8.861 **	2.575	2.278	2.190	0.252	4.322 *
Blue	0.337	0.077	0.229	7.315 *	0.314	2.366	0.690	0.001	3.498
W × B	1.759	3.300 *	0.485	4.631 *	1.176	3.735 *	5.990 **	0.496	3.222

Note: NSE. number of fruiting bodies; QR. quality mushroom rate; CH. cap hardness; CS. cap springiness; CC. cap chewiness; SH. stipe hardness; SS. stipe springiness; SC. stipe chewiness. * indicating significant difference level (*p* < 0.05). ** indicating significant difference level (*p* < 0.01).

**Table 4:** Two-way ANOVA (F-values) of the effects of white-light pretreatment and blue-light irradiation on nutritional quality of *P. pulmonarius* fruiting bodies.

Source	TS	ASH	MC	Fat	CF	CP	EAA	FAA
White	364.309 **	1333.390 **	11.842 **	1019.861 **	5324.803 **	1133.849 **	197.796 **	1365.215 **
Blue	332.655 **	348.344 **	9.519 **	46.536 **	104.875 **	617.278 **	2201.291 **	1051.004 **
W × B	491.094 **	258.95 **	0.937	329.204 **	1045.759 **	248.496 **	2378.108 **	1821.785 **

Note: TS. total sugar; MC. moisture content; CF. crude fiber; CP. crude protein; EAA. essential amino acids; FAA. free amino acid. ** indicating significant difference level (*p* < 0.01).

**Table 5:** Information entropy and weights of evaluation indicators for *P. pulmonarius* under different light regimes.

Indicator	*e*	*W*	Indicator	*e*	*W*
cap length	0.854	0.043	cap chewiness	0.816	0.055
cap width	0.891	0.032	stipe hardness	0.821	0.053
cap thickness	0.874	0.037	stipe springiness	0.886	0.034
stipe length	0.812	0.056	stipe chewiness	0.873	0.038
stipe diameter	0.898	0.030	total sugar	0.779	0.066
yield	0.805	0.058	ash	0.848	0.045
number of fruiting bodies	0.868	0.039	moisture	0.866	0.040
quality mushroom rate	0.891	0.033	fat	0.920	0.024
L*	0.887	0.033	crude fiber	0.879	0.036
a*	0.889	0.033	protein	0.820	0.053
b*	0.920	0.024	essential amino acids	0.927	0.022
cap hardness	0.814	0.055	free amino acid	0.903	0.029
cap springiness	0.892	0.032	Total		1

Note: *e*. Information entropy; *W*. Weight.

**Table 6:** Comprehensive evaluation results of *P. pulmonarius* under different light regimes based on entropy-weighted TOPSIS method.

Formula	D+	D−	C	Rank
W0B6	0.141	0.052	0.271	7
W0Bc	0.118	0.077	0.396	3
W4B6	0.139	0.065	0.320	4
W4Bc	0.138	0.058	0.297	5
W8B6	0.122	0.098	0.446	2
W8Bc	0.107	0.093	0.466	1
W12B6	0.157	0.055	0.261	8
W12Bc	0.141	0.058	0.292	6
CK	0.158	0.046	0.227	9

Note: D+, distance to the positive ideal solution; D−, distance to the negative ideal solution; C, relative closeness.

## Data Availability

The original contributions presented in the study are included in the article/Supplementary Material, further inquiries can be directed to the corresponding authors.

## Supplementary Materials

The following supporting information can be downloaded at: https://www.mdpi.com/article/10.3390/jof12060426/s1, Figure S1: Schematic diagram of the experimental setup. Shelves were placed against the wall, and each bag was positioned with its fruiting surface facing outward (parallel to the wall). LED light strips (white and blue) were installed directly facing the fruiting surface at a distance of 100 cm. Racks for different treatments were separated with blackout fabric to prevent light contamination.
