# Supplementary figures and images for: Effects of Dynamic Light Regimes on Yield and Quality Properties of Pleurotus pulmonarius Cultivar ‘Jinxiu’
Source: J Fungi (Basel). 2026 Jun 11;12(6):426. doi: 10.3390/jof12060426 (PMC13301140; doi:10.3390/jof12060426)

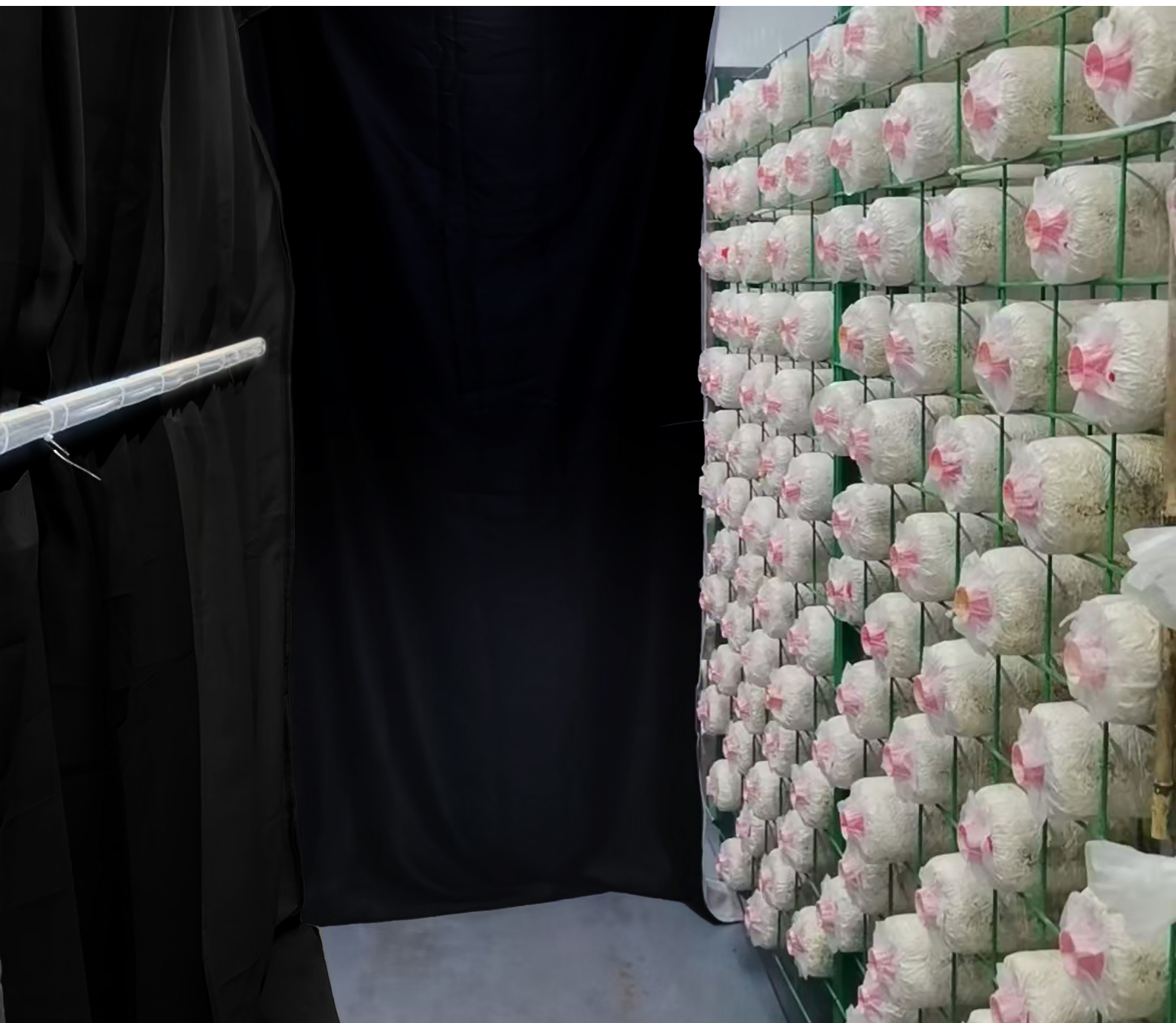

Supplement: Supplementary file 1 [file jof-12-00426-s001.zip › Figure S1.pdf]
